# The Identification of Recurrent Laryngeal Nerve by Injection of Blue Dye into the Inferior Thyroid Artery in Elusive Locations

**DOI:** 10.1155/2013/539274

**Published:** 2013-01-21

**Authors:** Gulcin Hepgul, Meltem Kucukyilmaz, Oguz Koc, Yigit Duzkoylu, Yavuz Selim Sari, Yesim Erbil

**Affiliations:** ^1^General Surgery Department, Bagcilar Training and Research Hospital, 34021 Istanbul, Turkey; ^2^General Surgery Department, Istanbul Training and Research Hospital, 34098 Istanbul, Turkey; ^3^General Surgery Department, Medical Faculty, Istanbul University, 34093 Istanbul, Turkey

## Abstract

*Introduction*. Thyroidectomy creates a potential risk for all parathyroid glands and the recurrent laryngeal nerve (RLN). The identification and dissection of the RLN is the gold standard for preserving its function. In some cases, it may be quite difficult to identify the nerve localization. In such elusive locations, we aimed to identify RLNs using peroperative injection of a blue dye into the inferior thyroid artery. *Materials and Methods*. This study included 10 selected patients whose RLN identification had been difficult peroperatively during the period from April 2008 to June 2009. When the RLNs became elusive in location, the branches of the inferior thyroid artery (ITA) on the capsule of the thyroid lobe were isolated, and then 0.5 mL isosulphan blue dye was injected into the artery. *Results*. RLN was carefully dissected in the tracheoesophageal groove. RLN was clearly visualized, in all patients. All RLNs were identified along their course in the dyed surrounding tissue. No RLN palsy was encountered. *Conclusion*. The injection of blue dye into the ITA branches can be used as an alternate method in case of difficulty in identification of RLNs.

## 1. Introduction

Thyroidectomy is one of the most frequent operations performed in iodine-deficient regions. The main postoperative complications are recurrent laryngeal nerve (RLN) palsy and hypoparathyroidism [1–3]. Although the overall incidence of nerve palsy is low, when it occurs, it becomes a devastating life-long handicap. The incidence of nerve palsy varies from 0% to 14%. Several factors influence the likelihood of injury to the nerve, including the underlying disease (substernal goiter, malignancy, Graves disease, etc.), the extent of resection, and the experience of the surgeon. The standard method for RLN preservation during thyroidectomy is routine visual identification of the nerve [1–3]. There are several approaches to identify the RLNs depending on the surgeon preference (distal or proximal to cricothyroid). But in some cases it may be still quite difficult to localize the RLNs, and in these situations, blue dye injection into the inferior thyroid artery or its branches may be useful to identify the RLN. To our best knowledge this is the first report of using peroperative injection of a blue dye into the inferior thyroid artery to identify the RLN.

## 2. Materials and Methods

**Figure 1:**
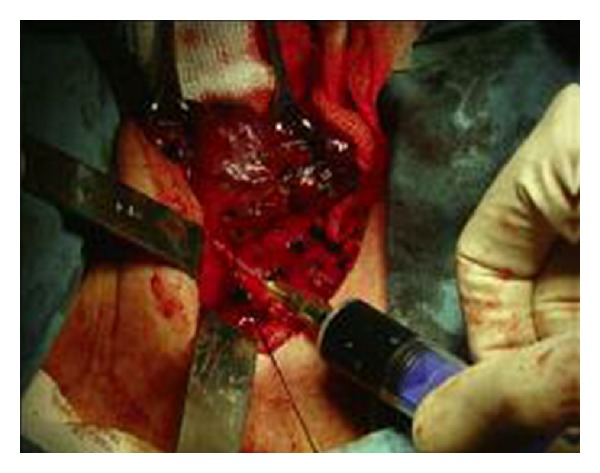
The injection of blue dye.

This study included 10 patients (8 women and 2 men) who were selected during the period from April 2008 to June 2009. The median age of the patients was 64 years (range 51–76 years). The indication for surgery was a large goiter with compressive effects in all patients. The study plan was reviewed and approved by our institutional ethics committee, and informed consent was obtained from all patients. Under general endotracheal anesthesia, the patients were placed in a supine position with the neck extended. A low collar incision was made and carried down through the subcutaneous tissue and platysma muscle. Superior and inferior subplatysmal flaps were developed, and the strap muscles were divided vertically in the midline and retracted laterally. The thyroid lobe was bluntly dissected free from its investing fascia and rotated medially. The middle thyroid vein was ligated. The superior pole vessels were ligated adjacent to the thyroid lobe. The RLNs were tried to identify in the tracheoesophageal groove. When decision made as an elusive location of RLN, the branches of the inferior thyroid artery (ITA) on the capsule of the thyroid lobe were isolated, and 0.5 mL of isosulphan blue dye was injected (Figure 1).

## 3. Results

**Figure 2:**
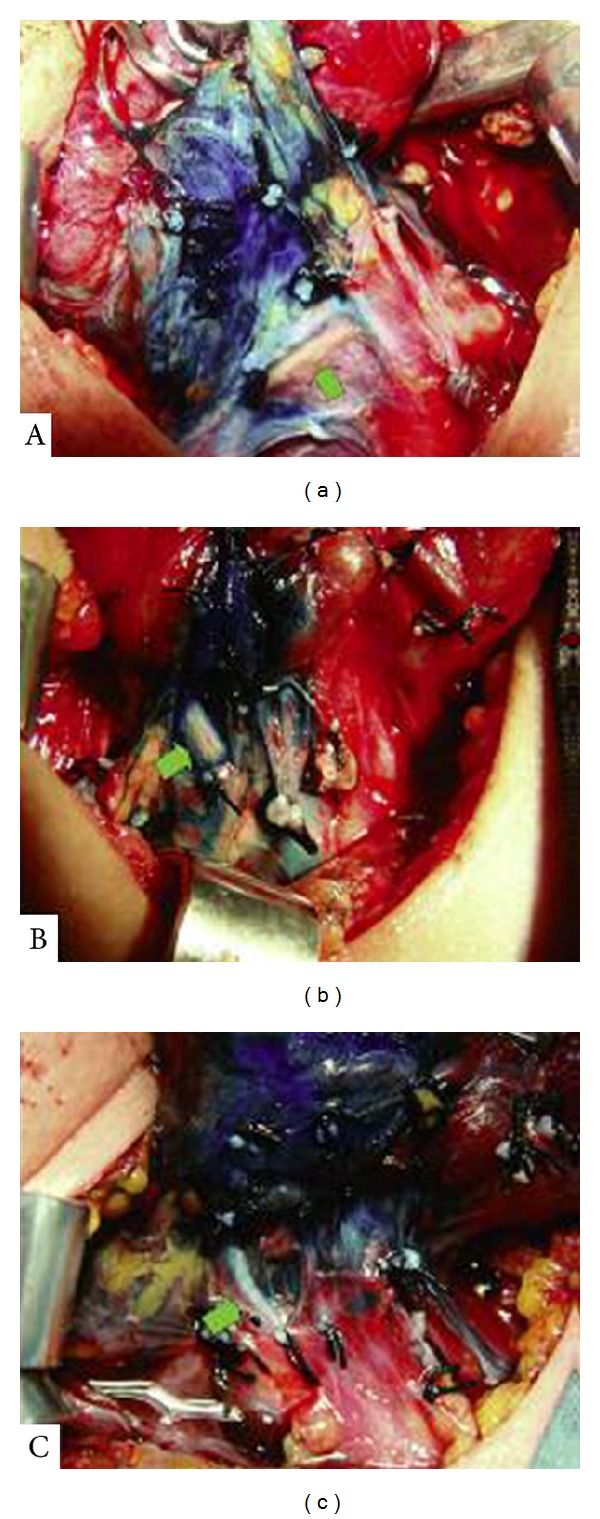
The nerve in the dyed surrounded tissue.

RLN was carefully dissected in the tracheoesophageal groove and clearly visualized in all the patients after injection of dye. All RLNs were identified along their course in the dyed surrounding tissue (Figures 2(a), 2(b), and 2(c)). The nerve was gently separated from its surrounding tissue. Once the nerve and parathyroid glands were identified and dissected, the thyroid lobe was removed from its tracheal attachments by dividing the ligament of Berry. The contralateral thyroid lobe was removed in a similar method. There was no operative mortality. Persistent or transient vocal cord paralysis was not encountered in any patients. In one (10%) patient, serum calcium level was found to be less than 8 mg/dL at the postoperative 24 hour. Papillary microcarcinoma was detected in 2 patients. There were no difficulties related to the blue dye injection during histopathological examination.

## 4. Discussion

Thyroidectomy creates a potential risk for the parathyroid glands and for the laryngeal nerves. Causes of RLN palsy include damage to the anatomic integrity of the nerve. Thermal lesions, difficulties in tracheal intubation leading to axon damage through excessive strain, edema or hematoma, and neuritis caused by scar tissue are some of the factors causing damage to the nerve structure. Neuritis as a result of viral infection may also damage the nerve. Galeno of Pergamo was the first anatomist to describe the RLN as a branch of a cranial nerve. In 1923, Lahey emphasized the importance of RLN and developed a standard technique for its identification and exposure during thyroidectomy [4, 5]. Since Lahey, identification and dissection of RLN is the gold standard of preserving its function. Identification of RLN has decreased the rates of transient or permanent nerve injury during thyroid operations [2, 3]. Many surgeons use relationships with the ITA, tracheoesophageal groove, and ligament of Berry as anatomical landmarks to identify the nerve. The first routine pattern for identifying the nerve is to find the inferior thyroid artery and to use it as an anatomic marker. However, because of the numerous variations of this neurovascular relationship altered also by the pathologic condition of the gland, identification of the artery does not assure consequent identification and preservation of the recurrent laryngeal nerve. If the nerve has not been found inferiorly, it is justifiable to search for it in the upper part of the gland using the posterior suspensory ligament of Berry as a landmark [6, 7]. Nevertheless, in some complicated cases or complementary thyroid surgery it may be very difficult to locate the RLNs; if you do not have another nerve identifying option such as neuromonitorization, you can easily use the dye method, and the RLN tract will be more definite. In conclusion, we propose that injection of blue dye into the inferior thyroid artery or its branches can be used as a method for the identification of the RLN. However, this technique should not be considered as a substitute for conventional visual identification of the nerve.
